# Concomitant MPZ and MFN2 Gene Variants and Charcot Marie Tooth Disease in a Boy: Clinical and Genetic Analysis—Literature Review

**DOI:** 10.1155/2022/3793226

**Published:** 2022-04-11

**Authors:** M. Comella, A. Collotta, V. Pavone, L. Ciccia, A. Bellinvia, C. Cerruto, M. G. L. Biondi, F. Pisani, P. Pavone

**Affiliations:** ^1^Postgraduate Training Program in Pediatrics, Department of Clinical and Experimental Medicine, University of Catania, Catania 95123, Italy; ^2^Department of General Surgery and Medical Surgical Specialties, Section of Orthopaedics and Traumatology, University Hospital Policlinico “Rodolico-San Marco”, University of Catania, Catania 95123, Italy; ^3^Child Neuropsychiatric Unit, University Hospital of Parma, Italy-Medicine & Surgery Department, Via Gramsci, 14, Parma 43126, Italy; ^4^Unit of Clinical Pediatrics, AOU “Policlinico”, PO “G.Rodolico”, University of Catania, Catania, Italy

## Abstract

Charcot- Marie- Tooth (CMT) disease includes a group of clinically and genetically heterogeneous neuropathic disorders with an estimated frequency of 1 on 2.500 individuals. CMTs are differently classified according to the age of onset, type of inheritance, and type of inheritance plus clinical features. For these disorders, more than 100 genes have been implicated as causal factors, with mutations in the *PMP22* being one of the most common. The demyelinating type (CMT1) affects more than 30% of the CMTs patients and manifests with motor and sensory dysfunctions of the peripheral nervous system mainly starting with slow progressive weakness of the lower extremities. We report here a 12 year- old boy presenting with typical features of CMT1 type, hearing impairment, and inguinal hernia who at the next-generation sequence analysis displayed a concomitant presence of two variants: the c.233 C>T p.Ser 78Leu of the *MPZ* gene (NM_000530.6) characterized as pathogenetic and the c.1403 G>A p.Arg 468His of the *MFN2* gene (NM_014874.3) characterized as VUS. Concomitant variant mutations in CMTs have been uncommonly reported. The role of these gene mutations on the clinical expression and a literature review on this topic is discussed.

## 1. Introduction

Inherited peripheral neuropathies include a group of disorders initially distinct in: Hereditary Motor and Sensory Neuropathy (HMSN), Hereditary Motor Neuropathy (HMN), Hereditary Sensory Neuropathy (HSN), and Autonomic Neuropathy (HSAN). Among this group of disorders, the HMSN, more commonly named Charcot-Marie-Tooth from the authors who first described the disorder, is the most common [1, 2]. Charcot-Marie-Tooth (CMT) encompasses a group of clinically and genetically heterogeneous disorders affecting the peripheral nervous system (PNS) with a prevalence inclusive of the total group of 1 in 2.500 individuals [1]. More than 100 different genes has been identified to cause the various forms [2]. CMTs may affect all period of life and so may manifest in infantile, childhood, juvenile, and in adult age. It may be inherited as autosomal dominant, autosomal recessive, or X-linked patterns. CMTs are classified according to the clinical expression and type of inheritance in CMT1 (demyelinating type), CMT2 (axonal type), CMT3 (Dejerine-Sottas disease), CMT4 (spinal type), CMT5 (pyramidal type), CMT6 (with optic atrophy), CMTDI (dominant intermediate type), CMTRI (recessive intermediate type); and CMTX (X-linked type). Each type of this group is further distinguished in several genetic subgroups according to the gene involved as causal factors [2–5]. Clinical features in each of these CMT types are various and complex but all clinically expressing with progressive weakness, wasting, and skeletal deformities.

We report here on a 12 year-old boy who showed a precocious clinical presentation of CMT1 with progressive peripheral weakness, and congenital bilateral inguinal hernia and neurosensorial deafness. The boy at the next-generation sequence analysis showed a concomitant presence of two variants: the c.233 C>T p.Ser 78Leu of the *MPZ* gene (NM_000530.6), characterized as pathogenetic and the c.1403 G>A p.Arg 468His of the *MFN2* gene (NM_014874.3), characterized as VUS. The role of each of these variants in the clinical expression of the disorder is discussed with a wide literature review.

## 2. Case Report

A 12-year-old boy came to the Unit of Clinical Pediatrics, Catania, Italy, for neurological checkup related to muscle weakness, worsening of his gait disturbance, and kyphoscoliosis. The family history as referred by the parents was extremely elusive as they only recalled that some relatives at the old age suffered of unclear gait disturbance. The neurological examination performed in the boy's parents (mother aged 38 and father 41 years old) and in the boy's twin sister was negative. The parents refused the genetic analysis.

The boy was born by a twin pregnancy, induced by intracytoplasmic sperm injection (ICSI). There is no familiarity between his parents. He was born at 34 weeks of gestation by cesarean section. At birth, he was admitted to a Neonatal Intensive Care Unit (NICU) for neonatal distress. During the hospitalization, he had surgery for bilateral inguinal hernia. The mother referred that the child had a motor development delay since his few years: he started to walk at 22 months with frequent falls and with a tendency to stepping gait and tiptoe. Cognitive development was normal. At the age of seven years, he got a cochlear implant due to bilateral sensorineural hearing loss (SNHL). At this age, the boy showed severe hypotrophic muscle masses especially of the lower limbs level with preserved muscular strength. He had scapular asymmetry, pectus excavatum, and kyphoscoliosis. The gait was abnormal with difficulty to walk on the heel to toe and tendency to walk on the tip, with a particular stepping gait. Bilateral pes cavus and a shortening of the left Achilles tendon was found. Fine touch was impaired. He was discharged with a diagnosis of CMT. One year later, the boy was newly readmitted to this institution. At the clinical examination, the boy is in a good general condition. His weight is 45 kg and the height 145 cm (all within normal limits for the age). Blood pressure is 100/70 mmHg. Cardiovascular and respiratory examinations are normal; spleen, and liver at normal limits. At neurological examination, he shows to be alert and well oriented with cranial nerves intact. Patellar tendon reflexes are hypo eligible. Touch, vibratory, and proprioceptive sensation slightly impaired. The peripheral nerve involvement is unchanged as well as skeletal deformities. A partial improvement of his hearing is reported. Laboratory tests including a complete blood count, electrolytes, coagulation testing, blood lactate, pyruvate, glucose, ketones, total cholesterol, CK, plasmatic purines and pyrimidines, plasma and urine amino acids, and urinary organic acids are normal. The spine X-ray confirms the kyphoscoliosis. The electrophysiological evaluation shows the absence of sensitive action potential (SAPs) and motor action potential (MAPs). Peroneal nerve conduction velocity is decreased (28 m/sec) with prolonged latency.

## 3. Search Procedure

The check was completed on three medical electronic databases (PubMed, Cochrane Library and Scopus Web of Science) by two authors (PP and MC) from 30 November 2019 till 30 December 2020. The search was focused on the type of mutation of the proband by searching the specific mutation of the *MPZ* and *MFN2* genes, as described in literature.

## 4. Selection Criteria and Data Extraction

Qualified studies for the present narrative review were performed exploring databases selecting with a screening of the titles and abstracts through the following inclusion criteria: time (publications in the last 10 years), language (written in the English language), and journal (studies published in specialized journals reporting clinical or pre-clinical results). Exclusion criteria were articles written in other languages, studies with no accessible data, or no accessible full text. We also excluded all of the remaining duplicates. The study selection and the data extraction were performed independently by authors (CL, CD, CA, CC, MC, BA, BM), and any divergences were resolved by discussion amongst the authors. The senior investigator (MC and PP) was consulted to revise the entire performance.

## 5. Materials and Methods

We selected from 19 articles published, nine papers, by selecting with the research of the mutation “c.233 C>T p.Ser78Leu of the gene *MPZ*,” and 10 papers, by selecting with the research of the other mutation “c.1403 G>A, p.Arg468His of the gene *MFN2*.” We selected only English articles, which contained performed studies mainly on pediatric population. We obtained and confirmed data by investigating each one author the validity of every single study. The study was approved from AOU Policlinico–Vittorio Emanuele from Catania with the protocol number VP0013442. CMT clinical and electrophysiological results of the probands and literature cases are reported presenting with *MPZ* and *MFN2* mutations.

## 6. Mutation Analysis of the Proband

Total genomic DNA of the proband was extracted from peripheral blood samples for the mutational analysis, following informed consent by the parents. WES analysis was performed using an Illumina TruSight One panel. The sample was sequenced by using the Illumina NexSeq 500 platform (Illumina Inc.) with 2 × 150 bp paired-end reads. Alignments and variants calls were generated using NextGene software (v2.4.1) and variant calls (with coverage <15X) were limited to the genes of interest.

## 7. Results

Genetic analysis at the Next Generation Sequence (NGS) carried up for peripheral neuropathy showed a concomitant two variants c.233 C>T p.Ser78Leu of the gene *MPZ* (NM_000530.6) and c.1403 G>A, p.Arg468His of the gene *MFN2* (NM_014874.3).

The former mutation is absent in the international databases of frequency (ExAC, GnomAD, 1000G), but it is described in LOVD3 and in ClinVar (ID14188) as pathogenic. It is noted in HGMD (CM941062) and described in literature (6) in association with autosomal dominant inheritance Charcot-Marie-Tooth 1B (MIM118200). It is also present a variant c.1403 G>A, p.Arg468His of the gene *MFN2* (NM_014874.3), reported as variant of uncertain significance (VUS) in ClinVar (ID2282: Benign; Like-Benign; Uncertain Significance) and in LOVD3 (pathogenic, benign, like-benign, VUS).

## 8. Discussion

Clinical features in CMTs show a wide intrafamilial and interfamilial expression as regard to the age of onset, progression of motor and sensory deficit, association with other clinical manifestations including ocular anomalies, cranial neuropathies, cognitive dysfunction, and dysautonomic disturbances [2–6]. CMTs usually start in the first two years of life with slow progressive impairment leading to distal muscle wasting and weakness, and in some cases, sensory loss and skeletal deformities. Clinical examination, electrophysiological findings, and genetic analysis are the best way to get a correct diagnosis including the single type of CMTs. The proband showed clinical features and electrophysiological findings, suggestive of the diagnosis of CMTs disease with onset of motor delay before of two years of age. He came first at our observation at the age of 12 years when the clinical features of peripheral neuropathy were evident manifesting with difficulty in walking and running, frequent falls, distal and symmetrical weakness and wasting, lower leg muscle hypotrophic, and pes cavus. In the course of one year, the gradually progressive course of the disease also involved the skeletal structure causing major evidence of kyphoscoliosis and of pectus excavatum. In the proband, the severe course and the results of the neurophysiological findings suggested the diagnosis of CMT type 1. Differentiation between CMT1 and CTM2 is not easy since both the two demyelinating and axonal types share some feature in common with the exception in the case of CMT2 of a less severe course of the symptoms and the findings of nerve conduction velocity which are usually not involved or only partially [6–8]. The proband showed two additional signs: congenital severe inguinal hernia that required a rapid surgical correction and a cranial neuropathy causing bilateral sensorineural hearing loss, which was treated with cochlear implantation.

Demyelinating neuropathies are linked to dysfunction of genes primarily affecting myelinating Schwann cells, while axonal neuropathies are linked to genes affecting mainly neurons and their long axons [1]. Among the wide number of genes implicated in the pathophysiologic mechanisms and various clinical manifestations of CMTs, over 90% of them have been related to four genes: the Peripheral Myelin Protein 22 (*PMP22*)- (OMIM ENTRY 601097); the Myelin Protein Zero (*MPZ*) (OMIM ENTRY 159440); the Gap Junction Protein Beta-1 (*GJB1*)- (OMIM ENTRY 304040), and the Mitofusin 2 (*MFN2*)-(OMIM ENTRY 608507) (9). *PMP22* is a large gene located in the middle of the Mb regions in chromosome 17p.12 [2]. Duplication or deletion in *PMP22* is cause of the disorder through a gene dosage effect [9]. *PMP22* related neuropathies comprise *PMP22* duplication leading to CMT disease type 1A; *PMP22* deletion causing hereditary neuropathy with liability to pressure palsies, and *PMP22* point mutations causing both phenotype [10]. The *GJB1*encodes for the ganglioside-induced differentiation associated protein 1, a member of the gap junction connexin family, membrane-spanning protein that assemble to form gap junction channels allowing the transfer of ions and small molecules between cells. In the Schwann cells, the protein is widely expressed and allows efficient transport connection between the outer and the interior myelin layers [11]. In a family with a novel mutation in the *GJB1* gene (p.Ser1281.eu) in both male and female a polyneuropathy with degree of severity from mild to severe was reported [11].

In the proband concomitant variants, c.233 C>T p.Ser78Leu of the gene *MPZ* and c.1403 G>A, p.Arg468His of the gene *MFN2* was reported. The *MPZ* gene maps to chromosome 1q22-q23 and encodes the most abundant peripheral nerve myelin protein. The MPZ protein acts as a hemophilic adhesion molecule in myelin compaction [12]. The final structure of MPZ protein consists of 248 amino acids disposed in three domains: extracellular domain comprising 124 amino acids; transmembrane domain comprising 26 amino acids, and intracellular domain comprising 69 amino acids located at the C-terminus [13]. Roa et al. [12] in 70 unrelated patients with demyelinating polyneuropathy for additional mutations in the *MPZ* gene by nucleotide sequence analysis identified a De novo mutation in *MPZ* exon 3 that predicts an Ile (135) Thr substitution in a family with clinically severe early onset CMT1. In a Finnish CMT patients study [14], screened for mutations in the peripheral myelin protein genes connexin 32 (*Cx32*), myelin protein zero *(MPZ*), and peripheral myelin protein 22 (*PMP22*), eleven Cx32 mutations in 12 families were identified, six with a CMT2 diagnosis, three with a CMT3 diagnosis and three with unclassified CMT. In the *MPZ* gene, a Ser78Leu mutation was found in one family with severe CMT1, and a de novo Tyr82Cys mutation in one patient with Dejerine-Sottas syndrome. Young et al. [15] report on a patient with a novel *MPZ* Glu141st op mutation presenting clinical features of axonal CMT2 with clinical features of the proband characterized by peroneal atrophy, foot deformities and atrophy of the intrinsic hand muscles, reduction of the Median Nerve Conduction Velocity (MNCV) of the ulnar nerve below 38 m/s and severe signs of peripheral neuropathy. They also found that clinical and electrophysiological heterogeneity among CMT patients carrying point mutations in *MPZ* and in *GJB1* is similar [15]. Sanmaneechai et al. [16] report on 103 patients from 71 families with 47 different *MPZ* mutations presenting a mean age of 40 years (range 3–84 years). The infantile onset group of patients had higher CMT neuropathy score (version 1 or 2) and slower nerve conduction velocity than in the other groups, the authors also noted that severity increased with the age. Kakar et al. [17] described a late onset CMT1type B associated to *MPZ* gene and a C-to-G transversion at nucleotide position 234, resulting in a serine-to-tryptophan mutation in codon 78 (S78W) of the translated protein. The Ser78Leu mutation caused in the patient bilateral, progressive numbness, tingling and weakness in his feet. Motor examination showed severe atrophy and weakness of the distal arm and leg musculature, with absence of patellar tendon reflexes. Electrophysiological studies showed the distal latencies were prolonged at twice normal and the compound muscle action potential amplitudes were severely reduced. Thirteen patients from 12 different families with eight different *MPZ* mutations were clinically evaluated by Shy et al. [18] in association with 64 cases of CMT1B collected by the literature and noted that patients may present with signs and symptoms of neuropathy both in early onset than later at the age of around 40 years. In a study performed in Japan [13], 64 CMT patients with *MPZ* variants were investigated. Most of them showed clinical signs prior of the age of 20 years. Cranial impairment was reported in 20 patients with dysarthria occurring in 7, dysphagia and hearing loss in 4, anisocoria, weakness of facial muscle, deviation of tongue protrusion in 3, nystagmus, strabismus, atrophy of facial muscle, atrophy of tongue in 2, and Adie's pupil, ptosis, sluggish light reflex, trigeminal neuralgia in single patient. In eight out 30 patients an elevated level of CK was also noted. The same authors [13] evaluating 85 patients with inherited peripheral neuropathy presenting with *MPZ* variants reported that the most frequent variants were: p.Arg98His, p.Thr124Met, p.Asp75Val, p.Arg98Cys, p.Asn35Tyr, and p.Ser78Leu. in contrast to *MPZ* variants reported in countries other than Japan in which the p.Ser78 Leu, p. His39Pro, p.Ser44Phe, p.Arg98His, p.Thr124Met, and p.Asp134Glu were the most frequent. In general, *MPZ* variants may clinically express with severe course and early onset to mild course starting in adult age. The *MPZ* variants have been associated to demyelinating neuropathy CMT1B and axonal neuropathy CMT2/j, the Dejerine-Sottas syndrome and congenital hypomyelinating neuropathy type 2 [13, 19], and dominant intermediate CMTDI [20].

*MFN2* is a gene located on the short (p) arm of chromosome 1 that encodes for Mitofuscin 2, a GTPase dynamin-like protein of the outer mitochondrial membrane: its role is predominant for several mitochondrial functions, including fusion, axonal transport, interorganellar communication, and mitophagy [21]. Mutations in the *MFN2* gene have been frequently associated with CMT disease type 2A (CMT2A). In a total of 73 unrelated patients with a diagnosis of CMT2, Engelfried et al. identified six novel mutations in *MFN2* gene: the c.380G>T (G127V), c.1128G>A (M376I), c.1040A>T (E347V), c.1403G>A (R468H), c.2113G>A (V705I), and c.2258_2259insT (L753fs) [22]. In a cohort study of Spanish patients with axonal CMT neuropathy, nine different *MFN2* mutations in 24 patients from 14 different families were found, all of mutations were present in heterozygous state. In this study, the *MFN2* mutations were related to CMT2 in 16% +/- that is 7% of the families studied and in 30.8 ± that is 14.2% (12/39) of families with known dominant inheritance [23]. Among 38 patients with CMT2 and 7 with Distal Hereditary Motor Neuropathy (DHMN), Luigetti et al. [24] found 6 patients with mutations in *MFN2*, 4 mutations in *HSPB1*, 2 mutations in *BSCL2*, 3 mutations in *GJB1*, and 1 mutation in *MPZ* [24]. Stuppia et al. [21] analyzed clinical, genetic, and neuropathological features and pathogenetic mechanisms associated to *MFN2* mutations. They maintain that in patients affected by CMTs with *MFN2* mutations a genotype-phenotype correlation is incomplete and also interfamilial clinical differences may be found. This is probably linked to environmental or other factors that modulate genetics. A complex case of *MFN2* p.R104W mutation has been reported by Genari et al. [25] who presented with severe and early onset axonal neuropathy, learning problems, obesity and glucose intolerance. Other features consisted of leukoencephalopathy, brain atrophy, and evidence of myelin involvement and changes of mitochondrial structure on sural nerve biopsy [25].

How *MFN2* mutations may produce degeneration to peripheral axons is a matter of debate. *MFN2* gene has been involved in several intracellular pathways that interact to regulate the mitochondrial network within neurons [21]. In a laboratory study, disease-associated *MFN2* mutant proteins induced abnormal clustering of small fragmented mitochondria in both neuronal cell bodies and proximal axons [26]. Complex *MFN2* molecular and cellular dynamics together with environmental and epigenetics factors may have a significant role [21].

Concomitant variant mutations have been uncommonly reported. Two mutations in *PMP22* gene 17p11.2-p12 duplication and a Ser72Leu point mutation were reported by Gouvea et al. [27] in a father and a daughter who had a very mild form of CMT disease. The author maintains that in the patients the effect more severe of one gene may be compensated by the functional effect of the other. Vital et al. [28] report a 41-year old woman and her daughter carrying a truncating mutation of *MFN2* (p.Val160fsX26) plus p.R120W in *GDAP1* variant who had a severe axonal form of CMT disease. The severe features of the patients have been related to a cumulative effect of *MFN2* and *GDAP1* mutation. A patient with severe axonal CMT and inherited heterozygous *MFN2* (p.Leu741Val) and *GDAP1* (p.Gln163) variants were reported by Fierro et al. [29]. A heterozygous double mutation in the *MPZ* gene including a “de novo” Val42 deletion and an Ala 221Thr substitution motherly inherited was identified in a patient affected by Dejerine-Sottas disease [30]. Two mutations in the *MPZ* Gene (His81Tyr and Val113Phe) were report in a 45-year-old female with a peripheral neuropathy with demyelinating and axonal features, pes cavus and pupillary light-near dissociation with a less severe phenotype in comparison to a patient with single His81 Arg mutation [31]. In a study carried in the Polish population, Drac et al. [32] described two CMT families in which the Ile135Thr and Pro132Leu mutations for the gene *MPZ* were identified. In the family carrying the Pro132Leu mutations, the sural nerve biopsy displayed hypomyelination-dysmyelination process.

In CMTs disease caused by concomitant variants the role of each gene leading to the clinical expression of the disorder is difficult to explain. The pathogenetic interaction of the concomitant mutations may have a double effect: antagonistic, each mutation prevailing on the other, or mixed both mutations interacting causing milder or more severe phenotypic features. In the proband *MPZ* and *MFN2* variant mutations were identified. *MPZ* has been associated to demyelinating neuropathy (CMT1B), to axonal neuropathy (CMT2/j), and juvenile-onset Dejerine-Sottas syndrome [13, 19] while *MFN2* is reported as primary cause of CMT disease type 2A [23]. The proband showed clinical and electrophysiological features of CMT type 1. In the proband is presumable to hypothesize that the effect of *MPZ* variant mutations prevailed on the *MFN2* variant mutations causing the more severe CMT type.

In addition to the classical signs of CMT disease type 1, the proband presented with congenital bilateral inguinal hernia and hearing loss due to cranial nerve involvement. Cranial nerve involvement are rarely observed in patients with CMT [13, 33]. Taniguchi et al. [13] among 66 CMT patients with *MPZ* variants reported the frequency of 3% of hearing loss similar to the frequency found in the normal population and suggest that the association *MPZ* variant and hearing loss could be questionable. Kobayashi et al. [34] report on a 23-year-old patient presented with progressive bilateral sensorineural hearing loss from 10 years of age who showed later neurological signs of CMTs. In the proband, the association of hearing loss and *MPZ* variant mutations is highly suggestive.

In conclusion, the present case shows that *MPZ* (c.233C<T) mutation gene may likely be the main pathogenetic cause of the CMT in this young boy and the concomitant presence of *MFN2* (c.1403G>) gene mutation could have contributed to the clinical expression. A clinical note: in a child, the positive test of auditory analysis skills must drive to a more thorough evaluation of the muscle condition as it may be a clue of possible CMT disorder and precocious treatment of the auditory dysfunction is mandatory.
